# Hampering Effect of Cholesterol on the Permeation of Reactive Oxygen Species through Phospholipids Bilayer: Possible Explanation for Plasma Cancer Selectivity

**DOI:** 10.1038/srep39526

**Published:** 2017-01-06

**Authors:** Jonas Van der Paal, Claudia Verheyen, Erik C. Neyts, Annemie Bogaerts

**Affiliations:** 1 Research group PLASMANT, Department of Chemistry, University of Antwerp, Universiteitsplein 1, B-2610 Wilrijk-Antwerp, Belgium

## Abstract

In recent years, the ability of cold atmospheric pressure plasmas (CAPS) to selectively induce cell death in cancer cells has been widely established. This selectivity has been assigned to the reactive oxygen and nitrogen species (RONS) created in CAPs. To provide new insights in the search for an explanation for the observed selectivity, we calculate the transfer free energy of multiple ROS across membranes containing a varying amount of cholesterol. The cholesterol fraction is investigated as a selectivity parameter because membranes of cancer cells are known to contain lower fractions of cholesterol compared to healthy cells. We find that cholesterol has a significant effect on the permeation of reactive species across a membrane. Indeed, depending on the specific reactive species, an increasing cholesterol fraction can lead to (i) an increase of the transfer free energy barrier height and width, (ii) the formation of a local free energy minimum in the center of the membrane and (iii) the creation of extra free energy barriers due to the bulky sterol rings. In the context of plasma oncology, these observations suggest that the increased ingress of RONS in cancer cells can be explained by the decreased cholesterol fraction of their cell membrane.

Over the last decades, cold atmospheric pressure plasmas (CAPs) have shown great potential in different fields, including, e.g., treatment of chronic wounds, sterilization of living and non-living surfaces or blood coagulation^1^,^2^. In this work, we focus on yet another field of study, which is called plasma oncology^3^,^4^. In this field, the use of CAPs as a new therapy for cancer treatment is explored. Indeed, both *in vitro* as well as *in vivo* studies illustrate that CAPs can be used to induce cell death in multiple cancer cell lines, including e.g., melanoma^5^,^6^,^7^,^8^,^9^,^10^,^11^,^12^, cervical^13^,^14^, lung^15^, breast^16^,^17^,^18^, glioblastoma^19^,^20^,^21^,^22^ and ovarian cancers^23^,^24^. Moreover, by tuning the plasma dose, which can be achieved by altering the intensity of the plasma or by changing the treatment time, plasmas can selectively induce cell death in cancer cells over healthy cells^8^,^18^,^22^,^25^,^26^. This could be a major improvement over the existing cancer treatment modalities. In fact, traditional therapies such as chemotherapeutic drug delivery still suffer from fundamental problems, including resistance as well as toxicity to normal cells^27^,^28^,^29^.

Although the selectivity of CAP treatment towards cancer cells is very promising, there is a clear need for fundamental insight into the underlying mechanisms^29^. Over the last years, multiple models have been proposed, but there is no consensus regarding the observed selectivity. All these models, however, stress the key role of reactive oxygen and nitrogen species (RONS) which are generated by the plasma^30^. Once the oxidative stress provoked by these reactive species exceeds the cellular antioxidative defense, signaling pathways which lead to cell death can be activated. Healthy cells are thought to deal better with this increased oxidative stress because they will take up less exogenous RONS and can neutralize these species more efficiently^31^. The main objective of this research is to provide new insights in the search for an explanation for this observed selectivity. Continuing upon the assumption that one of the major reasons behind the selectivity is that cancer cells absorb RONS faster compared to their healthy counterparts, a new question imposes itself: what difference between both cell types is responsible for this different uptake rate?

A possible explanation could be the increased expression of aquaporins (AQPs), a membrane protein family responsible for facilitating the diffusion of water across cellular membranes, in cancer cells. Indeed, it is shown that different cancer cell lines possess elevated levels of certain AQPs^32^,^33^,^34^,^35^. Breast cancer cell lines for example, show increased levels of AQP1, 4 and 5^32^,^33^, whereas AQP 1, 4, 8 and 9 are highly expressed in glioblastoma cell lines^33^,^34^,^35^. Due to the similarity of hydrogen peroxide (H_2_O_2_ - one of the most important RONS) and water, AQPs are also able to facilitate the passive diffusion of this reactive species through the plasma membrane^36^, which results in an increased oxidative stress. An important note, however, is that not all AQPs are able to transport H_2_O_2_ across the membrane equally efficiently^37^. This is due to the central pore present in each AQP, which acts as a selective filter. The central pore diameter of AQP1, for example, is only 2.8 Å which is too small to allow for an easy diffusion of H_2_O_2_, resulting in a very low permeability for H_2_O_2_ across this specific AQP^37^. AQP8, on the other hand, has a central pore diameter of 3.2 Å, enabling it to very efficiently transport H_2_O_2_^37^. The combination of the altered expression levels of certain AQPs together with the different central pore diameters of these AQPs could be used to explain the sensitivity of different cancer cell lines towards CAP treatment^38^.

In the present paper, however, we focus on yet another difference between the cell membrane of cancer cells and healthy cells, which is the cholesterol fraction of the membrane. It is known that cancer cell membranes contain lower levels of cholesterol compared to healthy cells^39^,^40^, which is an important difference since cholesterol is of great importance in maintaining the proper fluidity and rigidity in the plasma membrane of all animal cells^41^,^42^,^43^,^44^, in which it is one of the most abundant lipids with concentrations up to 50%^45^,^46^. Therefore, over the last years, the cholesterol fraction of cell membranes has been the subject of many computational^47^,^48^,^49^ and experimental^50^,^51^ investigations. These studies have provided a detailed understanding of the condensing and ordering effects of cholesterol. Due to these effects, an alteration of the cholesterol fraction might have a big effect on the permeation of RONS through the membrane of both cancer and healthy cells. In our previous work^52^ we showed that if lipid peroxidation (a process in which the impinging RONS oxidize the phospholipids of the membrane) occurs, a decreased cholesterol concentration will lead to a weakened membrane, eventually leading to pore formation, which did not occur when membranes contain elevated levels of cholesterol. Since the observed pore diameters were in the order of 15 Å, these pores could serve as channels through which H_2_O_2_, as well as other RONS, would be able to penetrate the membrane easily, leading to an increase of the intracellular oxidative stress. However, for these pores to be formed, oxidation degrees of over 60% were required^52^, at least on the simulated time scale. Since these high oxidation degrees might not be achievable during plasma treatments (no experimental data are available on the exact oxidation degree), the question arises what the effect of cholesterol on its own (without lipid peroxidation) would be on the permeation of different ROS through a lipid membrane. Therefore, in this study, molecular dynamics (MD) simulations are performed to calculate the potential of mean force (PMF) of H_2_O_2_, O_2_ (molecular oxygen), OH (hydroxyl radical) and HO_2_. (hydroperoxyl radical) across phospholipid membrane structures containing a varying cholesterol concentration, using the umbrella sampling (US) technique. This technique is used to improve sampling of the entire system, which would be hindered in a traditional MD simulation due to the presence of hydrophilic and hydrophobic regions in a phospholipid bilayer. From the obtained PMFs, free energy barriers for permeation across the bilayer systems can be derived. Molecular dynamics studies have been used frequently in the past in the field of plasma oncology and plasma medicine in general^53^. Several of these studies have shown the applicability of the US method. Indeed, Cordeiro used this method to calculate the free energy profiles of similar ROS through a POPC membrane and found a good agreement with experimental results for the permeation free energy barrier of H_2_O_2_^54^. Furthermore, Wennberg *et al*. used US simulations to investigate the effect of cholesterol on solute (e.g. ammonia, ethanol or benzene) partitioning into different phospholipid bilayer structures by varying the cholesterol concentration, lipid head group and lipid tail saturation^55^. One of their most important observations was the correlation between the area per phospholipid and the transfer free energy ΔG_w→tails_ for moving a solute from water into the lipid tail region, which was also in line with previous experimental results.

In the following sections, we will first describe how the membranes were constructed and simulated. Subsequently, the most important results are shown and the implications for plasma oncology are explained. It is important to note that similar reactive species as the ones investigated in this paper are also important in other, more traditional treatment methods^56^,^57^. Therefore, the research findings from this paper can thus be expanded to other oxidative stress inducing modalities such as radiation or e-beam therapy. However, CAP treatment is emphasized here due to the observed selectivity (as mentioned above).

## Simulation Methods

### Model systems

The model systems used in the simulations each contain in total 128 lipids. These lipids are either 1,2-dioleoyl-sn-glycero-3-phosphocholine (DOPC) or cholesterol. To examine the effect of the cholesterol concentration, 6 different systems were constructed which contain 0, 10, 20, 30, 40 or 50 mol% cholesterol. These concentrations were chosen to scan the full range of cholesterol fractions reported in literature for both cancer cells (23–32 mol%^39^,^40^) as well as healthy cells (38–50 mol%^39^,^40^,^45^). All membranes were generated using the Packmol package^58^ according to the following three rules:

- Cholesterol and DOPC molecules are equally divided over both bilayer leaflets.
- Within each leaflet, the lipids are randomly distributed in the xy-plane.
- To avoid unrealistically large forces at the beginning of the MD simulation, atoms from different lipids are separated by at least 2 Å.

The Packmol package is a software code that calculates the pairwise distance between any two atoms from different molecules and, iteratively, optimizes the structure until all molecules satisfy the above mentioned geometric constraints^58^.

**Figure 1:**
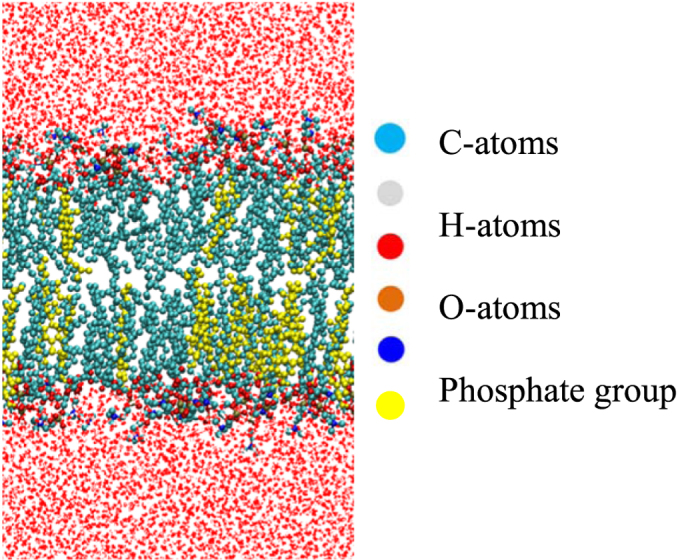
Structure of a bilayer containing 90 DOPC molecules (70 mol%), 38 cholesterol molecules (30 mol%) and 8000 water molecules. Phospholipids and water molecules are colored by atom type, whereas the cholesterol molecules are entirely colored in yellow, for the sake of clarity.

To create a hydrated membrane, the lipids were surrounded by 8000 water molecules (4000 in each leaflet). Each system was initially placed in a simulation box with dimensions 8 × 8 × 14 nm^3^. An example of one of the systems, containing 30 mol% cholesterol, is shown in Fig. 1.

### Simulation set-up

After constructing the hydrated bilayers, the systems were energy minimized using the steepest descent algorithm. Thereafter, the systems were equilibrated for 20 ns using the non-reactive united-atom GROMOS43A1-S3 force field^59^ for all lipids, with a time-step of 2 fs. Force field parameters for the different ROS that are investigated were taken from Cordeiro^54^. The simulations were performed in the NPT ensemble, using the Nosé-Hoover thermostat^60^ (with a reference temperature of 310 K and a coupling constant of 0.2 ps) together with the semi-isotropic Parrinello-Rahman barostat^61^ (with a reference pressure of 1 atmosphere, a compressibility of 4.5 × 10^−5^ bar^−1^ and a coupling constant of 1 ps). During these simulations, the coordinates and velocities of all atoms were listed every 100 ps for further use in the analysis of the membranes. Moreover, periodic boundary conditions were applied in all Cartesian directions. For the van der Waals interactions, a 1.0 nm cut-off was imposed, whereas for the electrostatics, the PME (Particle Mesh Ewald) method^62^,^63^ was used with 1.0 nm cut-off for real-space interactions and a 0.15 nm grid spacing for the reciprocal-space interactions, together with a fourth-order B-spline interpolation. All simulations, as well as the analysis, were carried out using the GROMACS 5.1 software package^64^.

### Umbrella sampling (US) simulations

The umbrella sampling technique, as originally developed by Torrie and Valleau^65^,^66^, uses the addition of an extra energy term, also called bias, to the system to ensure efficient sampling across an entire reaction coordinate. The sampling across the reaction coordinate can be executed in one simulation or in different simulations whose distributions overlap. These overlapping distributions are called the umbrella sampling windows. The exact mathematical description on how the bias is added, and how the unbiased free-energy differences are recovered afterwards, can be found elsewhere^67^.

**Figure 2:**
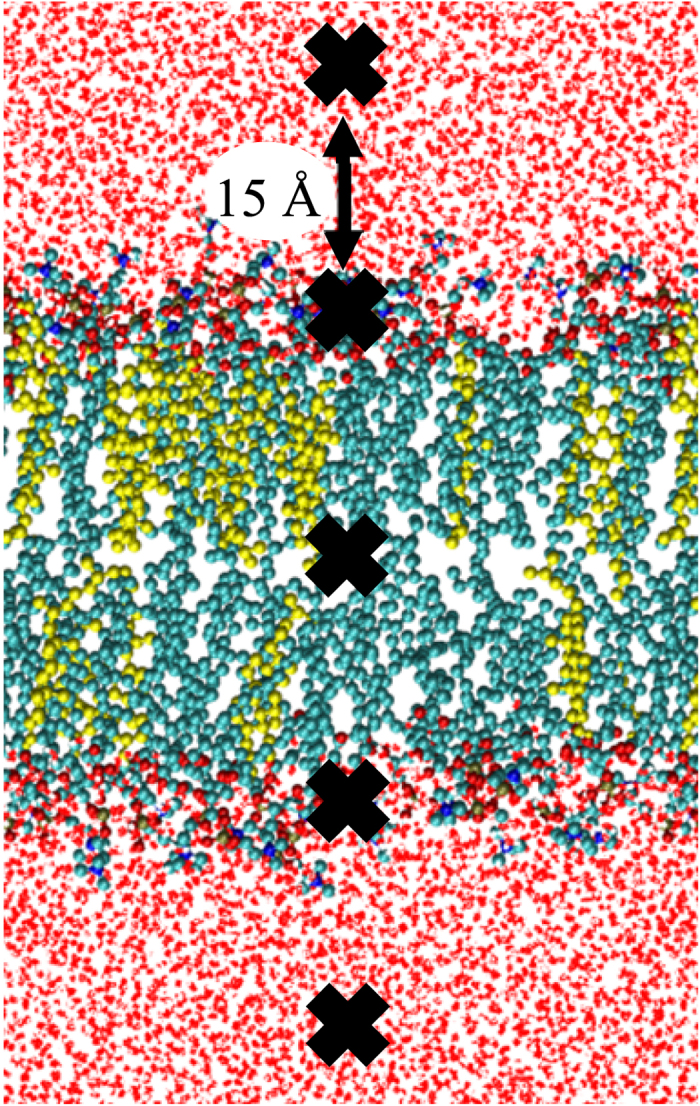
Illustration of the US simulation set-up. Five umbrella windows separated by 15 Å (position of ROS depicted by block crosses) are sampled in one simulation, thereby saving computational resources. In consecutive simulations, each species is shifted by 0.5 Å. To sample the entire membrane system, 30 simulations are performed, yielding a total of 150 umbrella histograms from which a PMF is constructed.

Starting structures for the US simulations were taken as the last frame of the equilibration. A total of 150 US windows were defined along the bilayer normal (i.e., the z-axis is the reaction coordinate in our systems), separated by 0.5 Å. As such, the sampling windows spanned the entire membrane system, starting from the lower water leaflet, crossing the lipid bilayer structure, and ending in the upper water leaflet. The umbrella windows were sampled in different simulations; however, to save computational resources, five umbrella windows were sampled during each simulation, keeping a distance of 15 Å between consecutive windows, which is illustrated in Fig. 2.

During these US simulations, reactive species were free to move in the xy-plane but their motion in the z-direction was restricted by applying a harmonic bias with a force constant of 1000 kJ.mol^−1^ nm^−2^. After an extra equilibration of 2 ns (same conditions as in the previous equilibration simulations), a 3 ns simulation was performed during which the US histograms were collected. PMFs were constructed by using a periodic version of the weighted histogram analysis method (WHAM)^68^, as implemented in the g_wham tool of GROMACS. The final PMFs shown in all figures following were obtained by averaging over 10 independent simulations (*i.e.*, starting from independently equilibrated structures).

### Analysis

To characterize the different membrane systems, and to be able to explain the observed trends in the PMFs, the (i) bilayer thickness, (ii) area per lipid, (iii) minimum interlipid distance and (iv) lipid density were calculated. The values shown below were calculated using the last 5 ns of each equilibration run.

The bilayer thickness was defined as the average distance along the z-axis between the COM of the phosphorus atoms of both leaflets. The area per lipid was calculated from the average box size (in the xy-plane), which was then divided by the number of lipids in one leaflet, *i.e.*, 64. This value gives thus an average area over both phospholipids and cholesterol. The minimum interlipid distance is defined as the smallest distance between two atoms from the lipid tails of different lipids (both phospholipids and cholesterol). Finally, for the calculation of the lipid density, the simulation box is divided in 100 slices (along the z-axis). Then, the average density of the lipid atoms in each slice is calculated.

## Results and Discussion

### Effect of ROS

**Figure 3:**
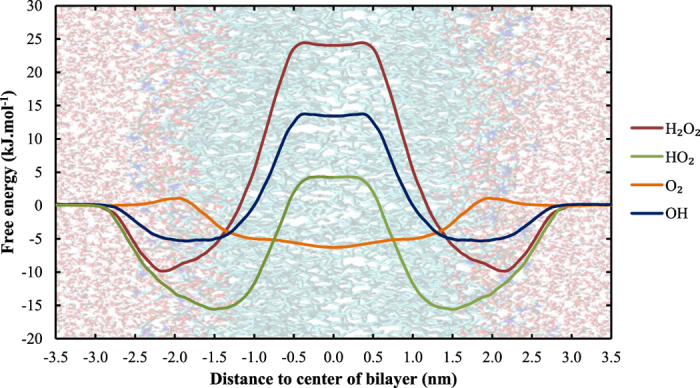
Free energy profile of different ROS across a pure DOPC membrane. The membrane structure is shown as a background to give an indication as to where the different regions (water layer, head group region, hydrophobic lipid core) are located.

To illustrate how the different reactive species behave when travelling through the bilayer, Fig. 3 shows the averaged PMFs for all ROS investigated across a pure DOPC membrane. Note that, although RNS are equally important in the field of plasma medicine, they could not be investigated in this study due to the lack of corresponding force field parameters of most RNS. However, in a non-reactive force field, we expect RNS to behave similar compared to ROS due to similarities in polarity and size. In fact, we have carried out some preliminary calculations for NO, for which parameters could be found, and the results show similar behavior between NO and O_2_. Table 1 contains the transfer free energy barriers associated with these profiles. Starting from the water layer, all hydrophilic ROS (H_2_O_2_, HO_2_ and OH) display an energy minimum at the phospholipid head group region, which originates from the stronger Coulombic interactions with these charged head groups. Consequently, due to the more polar character of this region (compared to the water layer), the hydrophobic O_2_-molecule shows a small increase in free energy.

Continuing towards the bilayer core, the role of the membrane as a permeation barrier can be derived from the increase in free energy of the hydrophilic ROS. O_2_ on the other hand displays an energy minimum in the center of the membrane, which will result in an accumulation of molecular oxygen in the bilayer core^69^. When comparing the transfer free energy barriers of all species, it is clear that the permeation of H_2_O_2_ is most hindered. The free energy barriers of HO_2_ and OH are very similar and there is almost no significant barrier for O_2_, which is in agreement with experimental evidence concerning the permeability of these different species^70^. The value of the free energy barrier of H_2_O_2_ (34.6 ± 3.3 kJ.mol^−1^) is in good agreement with other both computational (33 ± 4 kJ.mol^−1^)^54^ and experimental findings (36.8 kJ.mol^−1^)^71^. Furthermore, the significantly higher free energy barrier observed for H_2_O_2_ and the hydrophilic ROS can be explained by the number of hydrogen bonds these species can establish with surrounding water molecules. This number determines how easily the reactive species will lose their hydration shell upon penetrating into the membrane. Indeed, Cordeiro^54^ determined that H_2_O_2_ can establish twice as many H-bonds (combination of H-bonds as donor and acceptor gives a total of 4.5) compared to HO_2_ (2.3) and OH (2) radicals.

### Effect of cholesterol fraction

In the following sections, the effect of the cholesterol fraction is discussed for each of the species separately.

#### Hydrogen peroxide - H_2_O_2_

**Figure 4:**
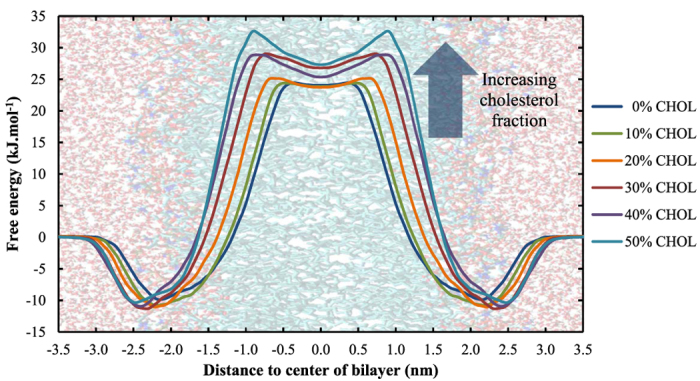
Effect of the cholesterol fraction in the cell membrane on the PMF of H_2_O_2_.

The free energy profiles of H_2_O_2_ across DOPC membranes containing a varying amount of cholesterol are shown in Fig. 4. The associated free energy barriers can be found in Table 2.

The membrane shown on the background is only an indication on where the different regions of the system are located. This is important to note because the bilayer thickness (and thus the position of the head groups etc.) depends on the cholesterol concentration (see Supporting Information, Figure S1A). This increasing bilayer thickness, which is accompanied by a decreasing area per lipid (Figure S1B), is known as the condensing effect of cholesterol^72^,^73^.

**Figure 5:**
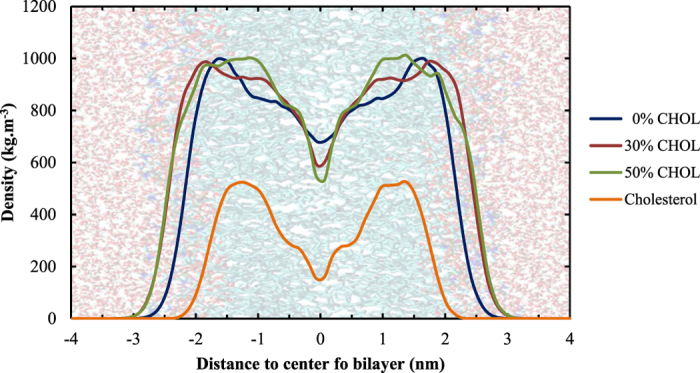
Total lipid density along the z-axis in membranes containing 0, 30 and 50% cholesterol, and the cholesterol density in systems containing 50% cholesterol.

Figure 4 shows that the cholesterol fraction has an influence on (i) the width and (ii) the height of the free energy barrier, and (iii) the shape of the PMF in the center of the bilayer. Because the minima of the PMFs are located around the head group region of the bilayer (see above), the width of the free energy barrier is a measure for the bilayer thickness. The variation in width can thus be explained by the increase in bilayer thickness (Fig. 5A). The consequence of this widening, however, is that the bilayer core is stabilized entropically at higher cholesterol concentrations, which will slow down the diffusion of H_2_O_2_ across the membrane.

A possible explanation for the increasing trend of the height of the free energy barrier can be found in the minimum interlipid distance, which is included in Table 2. Due to the condensing effect of cholesterol and the associated decreasing area per lipid, the minimum interlipid distance will decrease upon increasing the cholesterol fraction, as has been suggested in literature^74^. We assume that this will impede the penetration of H_2_O_2_ across the bilayer, which results in an increasing barrier. The last important observation that could be derived from Fig. 4, i.e., the appearance of a local minimum in the center of the bilayer when increasing the cholesterol fraction, can be explained by looking at the lipid density throughout the bilayer along the z-axis, which is plotted in Fig. 5.

**Figure 6:**
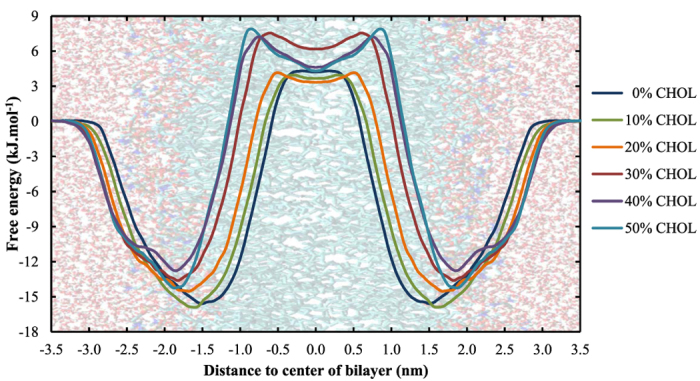
Effect of the cholesterol fraction in the cell membrane on the PMF of HO_2_.

Figure 5 illustrates that upon increasing the cholesterol fraction (i) the lipid density increases between z ≈ ±1.0–1.5 nm, while (ii) the lipid density decreases in the center of the bilayer. The increase around z ≈ ± 1.0–1.5 nm can be assigned to the bulky rings present in cholesterol (as can be derived from the cholesterol density profile, also plotted in Fig. 6), while the decrease in the center of the bilayer is due to the smaller structure of cholesterol (causing a depletion of lipids in the center). We thus assume that the combination of the increase in lipid density around the bulky rings together with the decrease in the center, are the underlying reason for the local minimum in the binary systems containing both phospholipids and cholesterol. Moreover, the higher the cholesterol fraction, the more pronounced is this local minimum (as can be derived from Fig. 4).

The increasing height of the energy barrier, combined with the appearance of a local minimum as well as the increased width of the barrier, will all contribute to hamper the penetration of H_2_O_2_ across membranes with higher cholesterol fractions. This is an important observation in the context of plasma oncology because the plasma membrane of cancer cells is known to possess lower amounts of cholesterol compared to healthy cells, which thus facilitates the penetration of H_2_O_2_, one of the most important ROS generated by CAPs, in these cells.

#### Hydroperxyl radical - HO_2_

The PMFs of HO_2_ across all simulated systems are shown in Fig. 6, with the associated transfer free energy barriers in Table 3.

In general, the effect of cholesterol on the PMF of HO_2_ is very similar compared to H_2_O_2_, i.e., (i) the energy barrier becomes wider and (ii) a local minimum appears in the center of the bilayer. The height of the free energy barrier is less affected compared to hydrogen peroxide, which is probably because HO_2_ is smaller than H_2_O_2_ making it less dependent on the decreasing interlipid distance. Moreover, we note that the depth of the energy minimum in the vicinity of the head groups decreases upon increasing the cholesterol fraction.

#### Hydroxyl radical - OH

**Figure 7:**
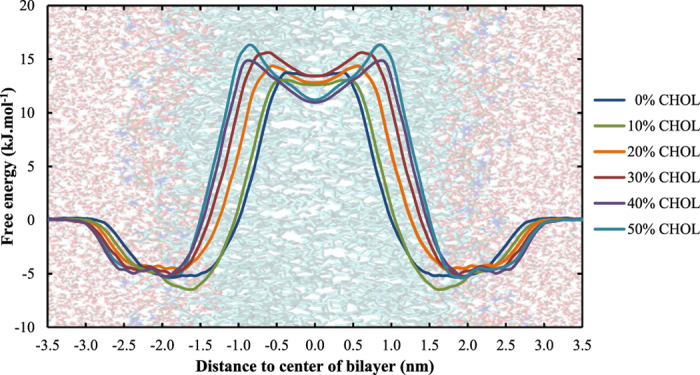
Effect of the cholesterol fraction in the cell membrane on the PMF of OH.

Figure 7 shows the PMFs of the transport of an OH radical through membranes containing a varying amount of cholesterol. The accompanying transfer free energy barriers are listed in Table 4. The general trends discussed in the sections above also appear in these graphs, i.e. an increasing barrier width and the formation of a local free energy minimum in the center of the bilayer. In contrast to HO_2_, the depth of the free energy minimum in the vicinity of the head group region is not affected by the cholesterol fraction in the case of an OH radical (in line with H_2_O_2_, see Fig. 4). The barrier height did not change significantly upon altering the cholesterol fraction, as was also the case for HO_2_ (see above).

#### Molecular oxygen - O_2_

**Figure 8:**
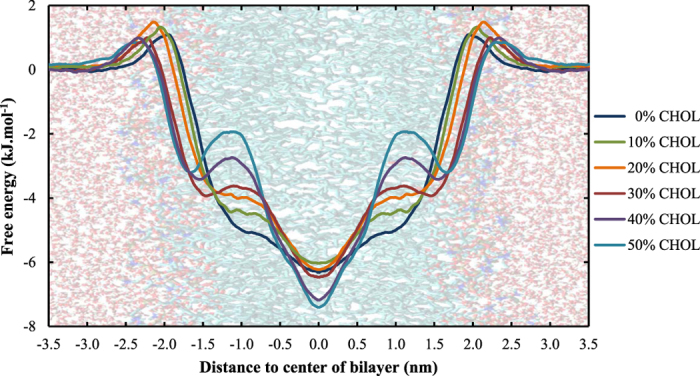
Effect of the cholesterol fraction in the cell membrane on the PMF of O_2_.

The free energy profiles of the last reactive oxygen species investigated, O_2_, are shown in Fig. 8. The exact values of the transfer free energy barrier are omitted since these barriers are not significant (as can be derived from Fig. 8). Due to the hydrophobic nature of this species, the PMFs are entirely different compared to all above (see discussion in Section 3.1). Upon increasing the cholesterol fraction (i) the free energy decreases in the center of the bilayer and (ii) a new barrier is formed at z ≈ 1.0 nm.

**Figure 9:**
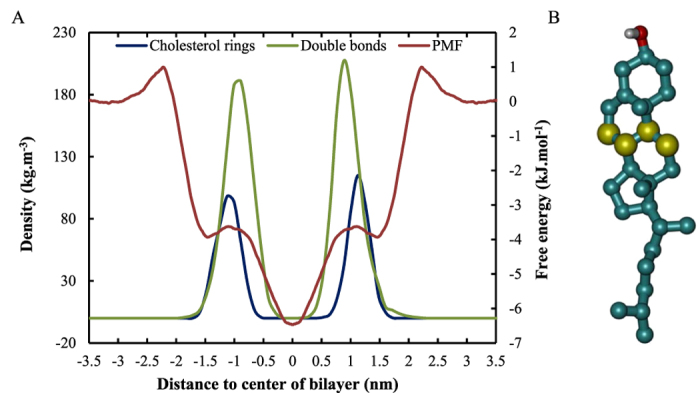
(**A**) Illustration of the overlap between the position of the cholesterol rings, the double bonds of DOPC and the extra free energy barrier for O_2_ in the bilayer around z = ±1.0 nm. The density of the carbon atoms which are shared between the second and third ring of cholesterol is plotted on the left axis, while the PMF of O_2_ across a 30:70 CHOL:DOPC membrane is plotted on the right axis. (**B**) The exact atoms of cholesterol whose density is plotted in A are depicted in yellow. These atoms are chosen since they are located in the middle of the bulky sterol rings.

The further decrease in free energy at the center of the bilayer will probably lead to an even more pronounced accumulation of O_2_ in the bilayer core of membranes with higher cholesterol concentrations. However, in the context of plasma oncology, the second trend, *i.e.*, the formation of a new energy barrier is probably more important. This new barrier is most likely caused by the bulky rings of cholesterol and the double bonds present in DOPC, which can be illustrated by comparing the location of these atoms in the membrane with the location of the extra barrier. This comparison is shown in Fig. 9 for the case of membranes containing 30% cholesterol.

The combination of the bulky rings and the double bonds make the region around z ≈ 1.0 nm the most rigid part of the entire membrane, due to which the penetration of O_2_ will be most hampered here. Although this new barrier is only a few kJ.mol^−1^ in height, it can be very important because, as mentioned before, O_2_ is required in the propagation step of the lipid peroxidation process. The extra free energy barrier in the vicinity of the double bonds, upon increasing cholesterol concentration, would lead to a depletion of O_2_ in this region and this can drastically lower the lipid peroxidation rate.

## Conclusion

United-atom molecular dynamics simulations were applied to investigate the effect of the cholesterol fraction in cell membranes on the permeation of different reactive oxygen species across these membranes. The results obtained in this study illustrate that, depending on the specific species, cholesterol is able to affect multiple aspects of the free energy profile, including (i) the height and (ii) width of the barrier, and (iii) the overall shape of the PMF (including the shape at the head group region, at the center of the membrane and at the sterol rings). Two of the most important shape-changing observations include (i) the appearance of local minima in the center of the bilayer in the free energy profiles of all hydrophilic ROS, which deepens upon increasing the cholesterol fraction, and (ii) the appearance of a free energy barrier at the double bond region in the PMF of O_2_, which increases when increasing the cholesterol fraction.

The appearance of a local minimum in the center of the bilayer is important because this will hamper the penetration of ROS towards the intracellular environment, even when the ROS succeed to penetrate into the membrane core.

Furthermore, although all PMFs shown indicate that cholesterol definitely has an influence on the penetration of certain ROS, even for systems without cholesterol, the free energy barriers are still significantly high for species to easily travel through the membrane, hence the permeation barrier role of each membrane. This leads to the conclusion that (i) either extra elements should be in play to explain the strong ingress of RONS during CAP treatment of cancer cells or that (ii) cancer cells are more vulnerable to the ingress of RONS due to their decreased capacity to reduce redox damage. One possibility to explain an increased ingress of RONS could be the expression of certain AQPs (see above). Another possibility would be the pores generated during the lipid peroxidation process, which will cause the membrane to lose its primary biological function (regulating transport from the extracellular to the intracellular matrix and vice versa), as illustrated in our previous work^52^. By breaching this barrier, RONS would be able to permeate into the cell, causing oxidative stress, which might lead to cell death. In this regard, the extra free energy barrier for O_2_ in the vicinity of the double bonds could be of great importance because it can drastically decrease the occurrence of this process. Indeed, one of the requirements for lipid peroxidation to occur is exactly the presence of O_2_ at the double bonds region. Consequently, in healthy cells, the ingress of RONS will be slowed down significantly due to the higher cholesterol fraction in their cell membrane.

In conclusion, our results provide a possible explanation as to why cancer cells, containing lower amounts of cholesterol, would absorb RONS faster compared to their healthy counterparts (containing higher levels of cholesterol), which is assumed to be one of the primary reasons behind the experimentally observed selectivity of CAP cancer treatment.

## Additional Information

**How to cite this article**: Van der Paal, J. *et al*. Hampering Effect of Cholesterol on the Permeation of Reactive Oxygen Species through Phospholipids Bilayer: Possible Explanation for Plasma Cancer Selectivity. *Sci. Rep.* **7**, 39526; doi: 10.1038/srep39526 (2017).

**Publisher's note:** Springer Nature remains neutral with regard to jurisdictional claims in published maps and institutional affiliations.

## Supplementary Material

Supplementary Information

## Figures and Tables

**Table 1:** Transfer free energies of all investigated ROS in a pure DOPC bilayer.

ROS	ΔG_water->tail_ (kJ.mol^−1^)
H_2_O_2_	34.6 ± 3.3
HO_2_	20.1 ± 2.3
OH	19.9 ± 2.8
O_2_	1.3 ± 0.4

The error bars are derived from the transfer free energies of all 10 independent profiles.

**Table 2:** Transfer free energy barrier of H_2_O_2_ in DOPC bilayers containing a varying fraction of cholesterol (column 2).

Cholesterol fraction (%)	ΔG_water->tail_ (kJ.mol^−1^)	Interlipid distance (Å)
0	34.6 ± 3.3	3.063 ± 0.002
10	36.3 ± 2.9	2.926 ± 0.018
20	36.7 ± 3.0	2.772 ± 0.012
30	40.8 ± 4.1	2.660 ± 0.035
40	40.5 ± 3.7	2.589 ± 0.036
50	43.4 ± 3.8	2.558 ± 0.046

Column 3 shows the averaged minimal interlipid distances in these systems.

**Table 3:** Transfer free energy barrier of HO_2_ in DOPC bilayers containing a varying fraction of cholesterol.

Cholesterol fraction (%)	ΔG_water->tail_ (kJ.mol^−1^)
0	20.1 ± 2.3
10	20.8 ± 3.1
20	19.4 ± 2.2
30	21.9 ± 2.7
40	21.0 ± 3.0
50	23.2 ± 2.8

**Table 4:** Transfer free energy barrier of OH in DOPC bilayers containing a varying fraction of cholesterol.

Cholesterol fraction (%)	ΔG_water->tail_ (kJ.mol^-1^)
0	19.9 ± 2.8
10	20.4 ± 3.2
20	19.9 ± 2.8
30	22.3 ± 2.1
40	21.1 ± 2.3
50	22.1 ± 2.4
