# Supplementary material for: Hampering Effect of Cholesterol on the Permeation of Reactive Oxygen Species through Phospholipids Bilayer: Possible Explanation for Plasma Cancer Selectivity
Source: Sci Rep. 2017 Jan 6;7:39526. doi: 10.1038/srep39526 (PMC5216346; doi:10.1038/srep39526)
Supplement: Supplementary Information [file srep39526-s1.pdf]

SUPPORTING INFORMATION

HAMPERING EFFECT OF CHOLESTEROL ON THE PERMEATION OF REACTIVE OXYGEN SPECIES THROUGH PHOSPHOLIPIDS BILAYER: POSSIBLE EXPLANATION FOR PLASMA CANCER SELECTIVITY

Jonas Van der Paal, Claudia Verheyen, Erik C. Neyts and Annemie Bogaerts(*)

Research group PLASMANT, Department of Chemistry, University of Antwerp, Universiteitsplein 1, B-2610 Wilrijk-Antwerp, Belgium

(*) Corresponding author (e-mail address: annemie.bogaerts@uantwerpen.be)

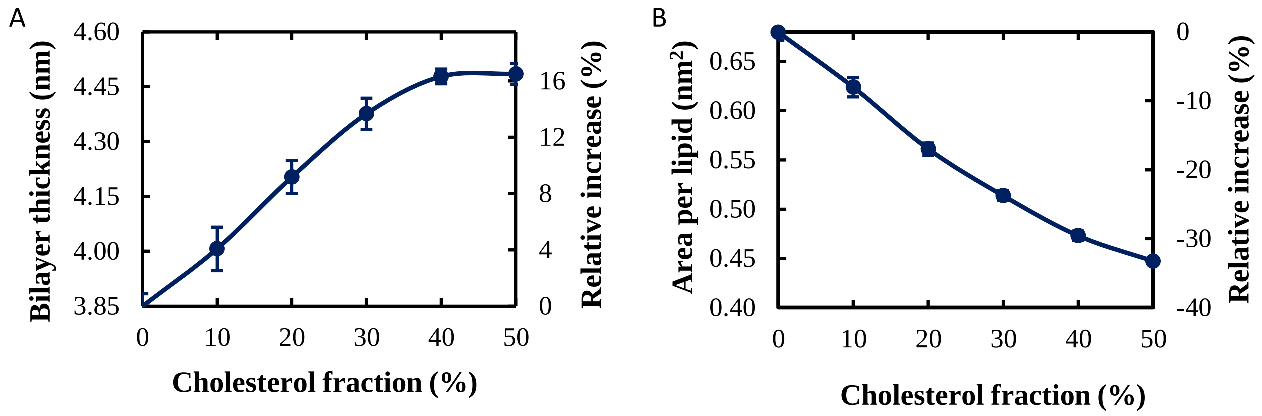

Figure S1: Bilayer thickness (A) and area per lipid (B) as a function of the cholesterol concentration of the membrane. Absolute values are shown on the left axis, while relative values (with respect to the non-oxidized system) are depicted on the right axis.
